# Methylene blue inhibits NLRP3, NLRC4, AIM2, and non-canonical inflammasome activation

**DOI:** 10.1038/s41598-017-12635-6

**Published:** 2017-09-29

**Authors:** Huijeong Ahn, Seung Goo Kang, Sung-il Yoon, Hyun-Jeong Ko, Pyeung-Hyeun Kim, Eui-Ju Hong, Beum-Soo An, Eunsong Lee, Geun-Shik Lee

**Affiliations:** 1 College of Veterinary Medicine and Institute of Veterinary Science, Kangwon National University, Chuncheon, Gangwon 24341 Republic of Korea; 2 Department of Molecular Bioscience, School of Biomedical Science, Kangwon National University, Chuncheon, Gangwon 24341 Republic of Korea; 3 Division of Biomedical Convergence, College of Biomedical Science, Kangwon National University, Chuncheon, Gangwon 24341 Republic of Korea; 4 Laboratory of Microbiology and Immunology, College of Pharmacy, Kangwon National University, Chuncheon, Gangwon 24341 Republic of Korea; 5 College of Veterinary Medicine and Institute of Veterinary Science, Chungnam National University, Daejeon, 34134 Republic of Korea; 6 Department of Biomaterial Science, College of Natural Resources and Life Science, Pusan National University, Gyeongsangnam-do, 50612 Republic of Korea

## Abstract

Methylene blue (MB), which has antioxidant, anti-inflammatory, neuroprotective, and mitochondria protective effects, has been widely used as a dye and medication. However, the effect of MB on inflammasome activation has not yet been studied. Inflammasomes are multi-protein complexes that induce maturation of interleukins (ILs)-1β and -18 as well as caspase-1-mediated cell death, known as pyroptosis. Dysregulation of inflammasomes causes several diseases such as type 2 diabetes, Alzheimer’s disease, and gout. In this study, we assess the effect of MB on inflammasome activation in macrophages. As the result, MB attenuated activation of canonical inflammasomes such as NLRP3, NLRC4, and AIM2 as well as non-canonical inflammasome activation. In addition, MB inhibited upstream signals such as inflammasome assembly, phagocytosis, and gene expression of inflammasome components via inhibition of NF-κB signaling. Furthermore, MB reduced the activity of caspase-1. The anti-inflammasome properties of MB were further confirmed in mice models. Thus, we suggest that MB is a broad-spectrum anti-inflammasome candidate molecule.

## Introduction

Methylene blue (MB, 3,7-bis(dimethylamino)-phenothiazin-5-ium chloride) is a heterocyclic aromatic chemical compound with the chemical formula C_16_H_18_N_3_SCl^1^. It has many uses in biology and chemistry, such as a dye for the textile industry, and has potent antibiotic and antioxidant properties. Since its discovery as the first synthetic anti-malarial agent by Ehrlich in 1891, MB has been used in several clinical fields for the treatment of acute and chronic methemoglobinemia, carbon monoxide poisoning, urinary tract infection, septic shock, and cardiopulmonary bypass^1^. MB suppresses production of superoxide radicals by acting as an alternative receptor of xanthine oxide electrons. Recently, MB has received increased attention in view of studies suggesting its usefulness in treating mitochondrial dysfunction^1^. It has also been studied as an agent for the treatment of Alzheimer’s disease^2^. MB may also provide neuroprotective functions based on its anti-inflammatory properties^3^. Further, expression of inflammatory genes was reduced in microglia treated with lipopolysaccharide (LPS) in the presence of MB in the culture media^3^.

Inflammation is a protective immune response mediated by the innate immune system in response to harmful stimuli such as pathogens, damaged cells, and irritants and is tightly controlled by the host^4^. Insufficient inflammation can cause continuous infection of pathogens, whereas excessive inflammation can lead to chronic or systemic inflammatory diseases. Innate immune function depends on germline-encoded pattern-recognition receptors (PRRs) recognizing pathogen-associated molecular patterns (PAMPs) derived from infectious pathogens as well as danger-associated molecular patterns (DAMPs) induced from endogenous stress. Inflammasomes, which are multi-protein complexes, consist of cytosolic PRRs and sensing cytosolic PAMPs or DAMPs in myeloid cells as well as non-myeloid cells such as keratinocytes, hepatocytes, and cardiomyocytes^5–10^. To assemble the inflammasome complex, sensing proteins and caspase-1 are linked by an adaptor protein known as apoptosis-associated speck-like protein containing a carboxy-terminal caspase recruitment domain (Asc or pycard). The sensing proteins are nucleotide-binding oligomerization domain (NOD), leucine-rich repeat (LRR)-containing protein (NLR) family members such as NLRP1, NLRP3, and NLRC4, or absent in melanoma 2 (AIM2)^5,6^. Upon detecting certain stimuli, NLR or AIM2 can oligomerize into a caspase-1-activating scaffold. Active caspase-1 subsequently functions to cleave the proinflammatory IL-1 family of cytokines into their bioactive forms, IL-1β and IL-18, as well as induce pyroptosis, a type of inflammatory cell death^5^. In addition, it has been suggested that the non-canonical inflammasome activates caspases-4, -5, and/or -11 in response to intracellular LPS, resulting in IL-1β/-18 secretion, pyroptosis, and endotoxemic death^11^.

Although MB has been used in human and veterinary medicine for over a century, there has been no study on the role of MB on inflammasome activation. In this study, we assessed the effect of MB on several well-characterized inflammasomes such as NLRP3, NLRC4, and AIM2 and non-canonical inflammasomes in murine macrophages. In addition, we demonstrated the upstream and molecular mechanisms of MB in the context of inflammasome activation. The regulatory effect of MB were further confirmed with animal models. Thus, we conclude that MB regulates inflammasome activation and inflammatory responses.

## Results

### Methylene blue inhibits NLRP3 inflammasome activation

**Figure 1:**
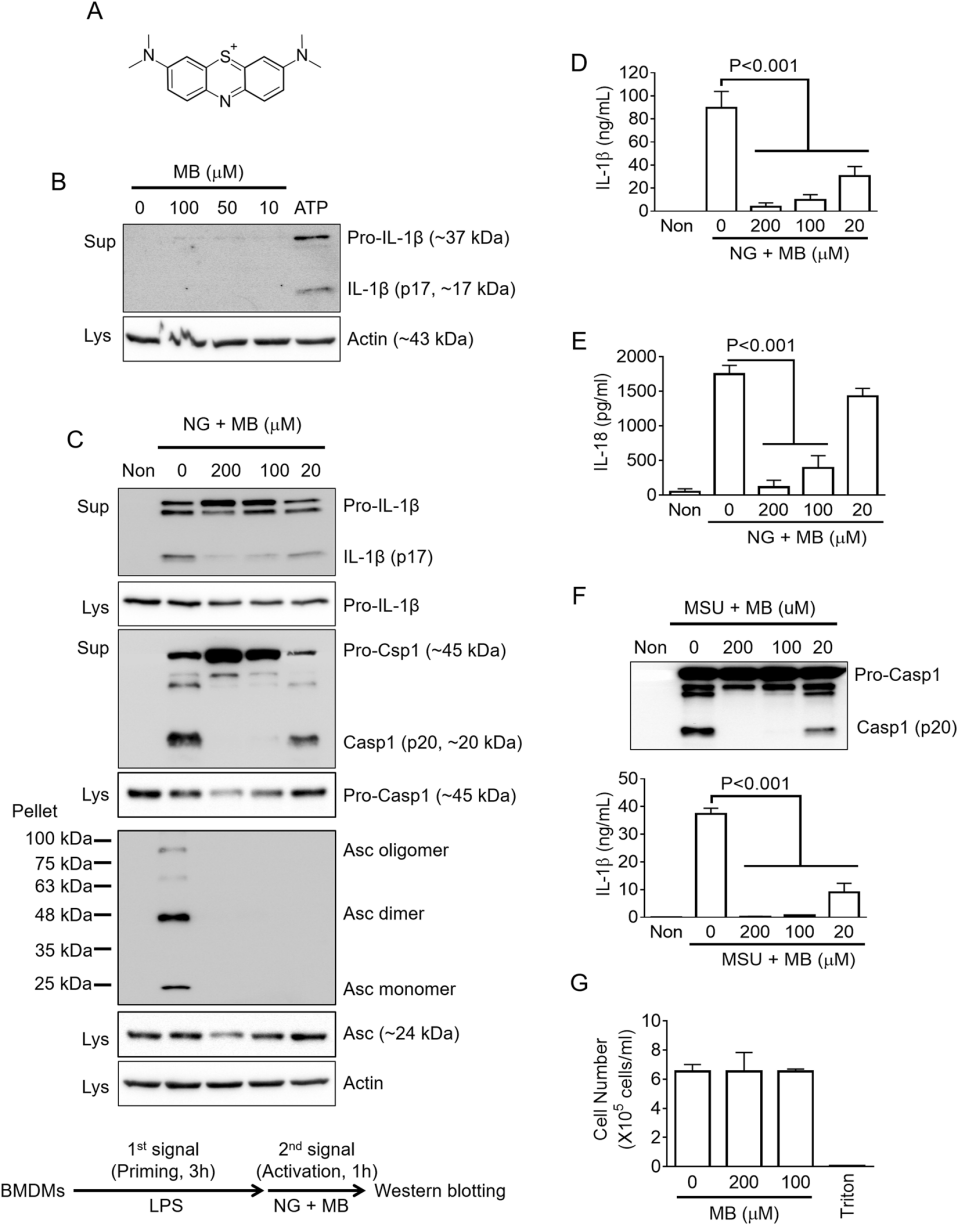
Effect of methylene blue on NLRP3 inflammasome activation. (**A**) Chemical structure of methylene blue (MB). (**B**) Lipopolysaccharide (LPS)-primed bone marrow-derived macrophages (LPS-primed BMDMs) were treated with the indicated concentration of MB or ATP (2 mM) as a positive control. Secretion of active form of IL-1β was analyzed by immunoblotting using cell culture supernatants (Sup) and cell lysates (Lys). (**C**–**E**) LPS-primed BMDMs were treated with the indicated dosage of MB with/without nigericin (NG, 40 μM). (**C**) Secretion of IL-1β and caspase-1 (Casp1) and formation of Asc pyroptosome were analyzed by immunoblotting using Sup, Lys, and cross-linked pellets (Pellet) from whole cell lysates. The below schematic graph displays the chemical treatment process for inflammasome activation. IL-1β (**D**) and IL-18 (**E**) secretions were measured by ELISA. (**F**) LPS-primed BMDMs were treated with monosodium urate crystals (MSU, 800 μg/mL). Secretion of caspase-1 was analyzed by immunoblotting, and IL-1β secretion was measured by ELISA. (**G**) For cytotoxicity, BMDMs were treated the indicated dosages of MB, and cell number was measured by an automated cell counter. Triton x-100 (1%, Triton) treatment led to cell death. All immunoblot data shown are representative of at least three independent experiments. Bar graph presents the mean ± SD.

To assess the effect of methylene blue (MB, Fig. 1A) on IL-1β maturation resulting from inflammasome activation, LPS-primed bone marrow-derived macrophages (BMDMs) were treated with MB or ATP, a NLRP3 inflammasome trigger, as a positive control. Although ATP treatment induced IL-1β secretion resulting from NLRP3 inflammasome activation, MB alone did not (Fig. 1B). This result implies that MB alone did not activate inflammasomes. Next, we tested whether or not MB inhibits inflammasome activation. LPS-primed BMDMs were subjected to NLRP3 inflammasome activation by nigericin (NG) or ATP in the presence of increasing dosages of MB, and several readouts for inflammasome activation were observed such as secretion of maturated IL-1β and caspase-1, as well as formation of Asc pyroptosome (Fig. 1C and Supplementary Fig. 1). As the result, MB dose-dependently attenuated secretion of IL-1β (p17) and caspase-1 (p20) as well as aggregation of Asc. In addition, we confirmed the anti-inflammasome properties of MB on NG-induced IL-1β (Fig. 1D) and IL-18 (Fig. 1E) secretion by ELISA. These data suggest that MB inhibited assembly of the NLRP3 inflammasome, which is upstream of caspase-1, IL-1β, and IL-18 maturation. Furthermore, MSU crystals, another NLRP3 inflammasome trigger, induced caspase-1 and IL-1β secretion, which were blocked by MB treatment (Fig. 1F). The current concentration of MB did not present any cytotoxicity in BMDMs (Fig. 1G). Taken together, we suggest that MB is a putative anti-NLRP3 inflammasome agent.

### Methylene blue interrupts gene expression of NLRP3 and cytokines

**Figure 2:**
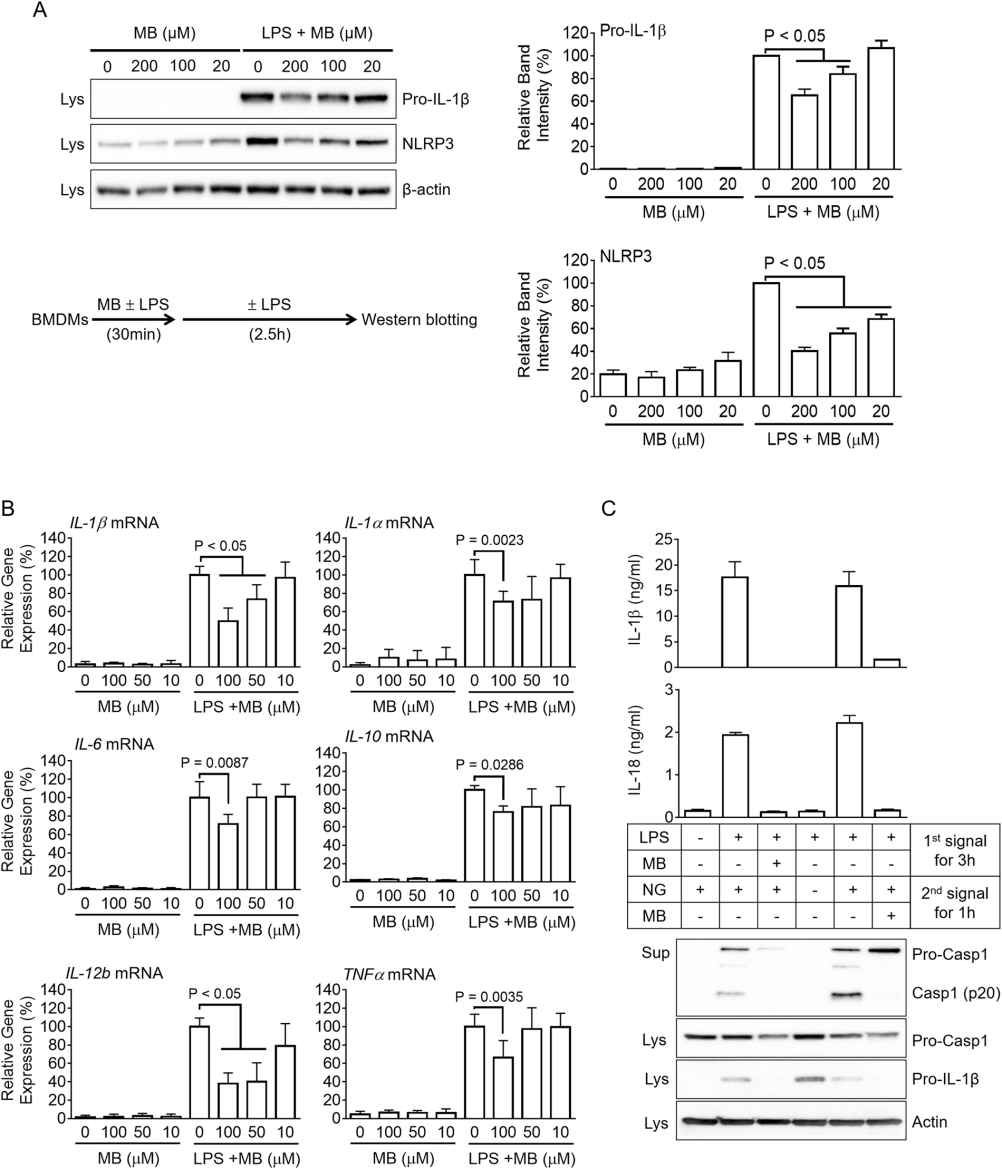
Effect of MB on the priming step of inflammasome activation and expression of other cytokine genes. (**A** and **B**) BMDMs were treated with the indicated concentration of MB with/without LPS (10 ng/mL) as indicated in the schematic graph. (**A**) Pro-IL-1β and NLRP3 expression levels were analyzed by immunoblotting and further presented with band density. (**B**) Expression levels of mouse *IL-1β, IL-1α, IL-6, IL-10, IL-12b*, and *TNFα* mRNAs were quantitated by real-time PCR. **C**, BMDMs were treated with MB and/or LPS as the 1^st^ signal, after which cells were replaced by media containing nigericin (NG, 2^nd^ signal) with/without MB as the 2^nd^ signal. IL-1β and IL-18 secretion levels were measured by ELISA, and Casp1 secretion and pro-IL1β expression were analyzed by immunoblotting. All immunoblot data shown are representative of at least three independent experiments. Bar graph presents the mean ± SD.

NLRP3 inflammasome activation requires a priming step in which toll-like receptor (TLR) ligands such as LPS induce production of the pro-forms of IL-1β and NLRP3^12,13^. To elucidate the effect of MB on the priming step, BMDMs were treated with MB with/without LPS (Fig. 2A). MB alone did not have any effect on gene expression, although it interrupted LPS-mediated pro-IL-1β and NLRP3 production. This result suggests that MB inhibits NLRP3 inflammasome activation as well as the priming step. In addition, we elucidated the effect of MB on the mRNA expression of other cytokines such as *IL-1α, IL-6, IL-10, IL-12b*, and *TNFα* in BMDMs (Fig. 2B). In the results, MB interrupted up-regulation of cytokines in response to LPS treatment, implying that MB attenuates the LPS-TLR4 signaling pathway. We next investigated whether or not MB inhibits both the priming and activation of inflammasomes. BMDMs were separately treated with MB at the 1^st^ or 2^nd^ steps (Fig. 2C). MB treatment at the 1^st^ step blocked secretion of IL-1β, IL-18, and caspase-1, implying that MB interrupts priming of inflammasome activation. In addition, co-treatment of MB with NG, as a 2^nd^ signal trigger, did not result in maturation of IL-1β, IL-18, and caspase-1. Taken together, MB interrupts both the priming and activation of inflammasomes as well as cytokine expression.

### Methylene blue attenuates mitochondrial ROS production, phagocytosis, caspase 1 activity, and NLRP3 promoter activity

**Figure 3:**
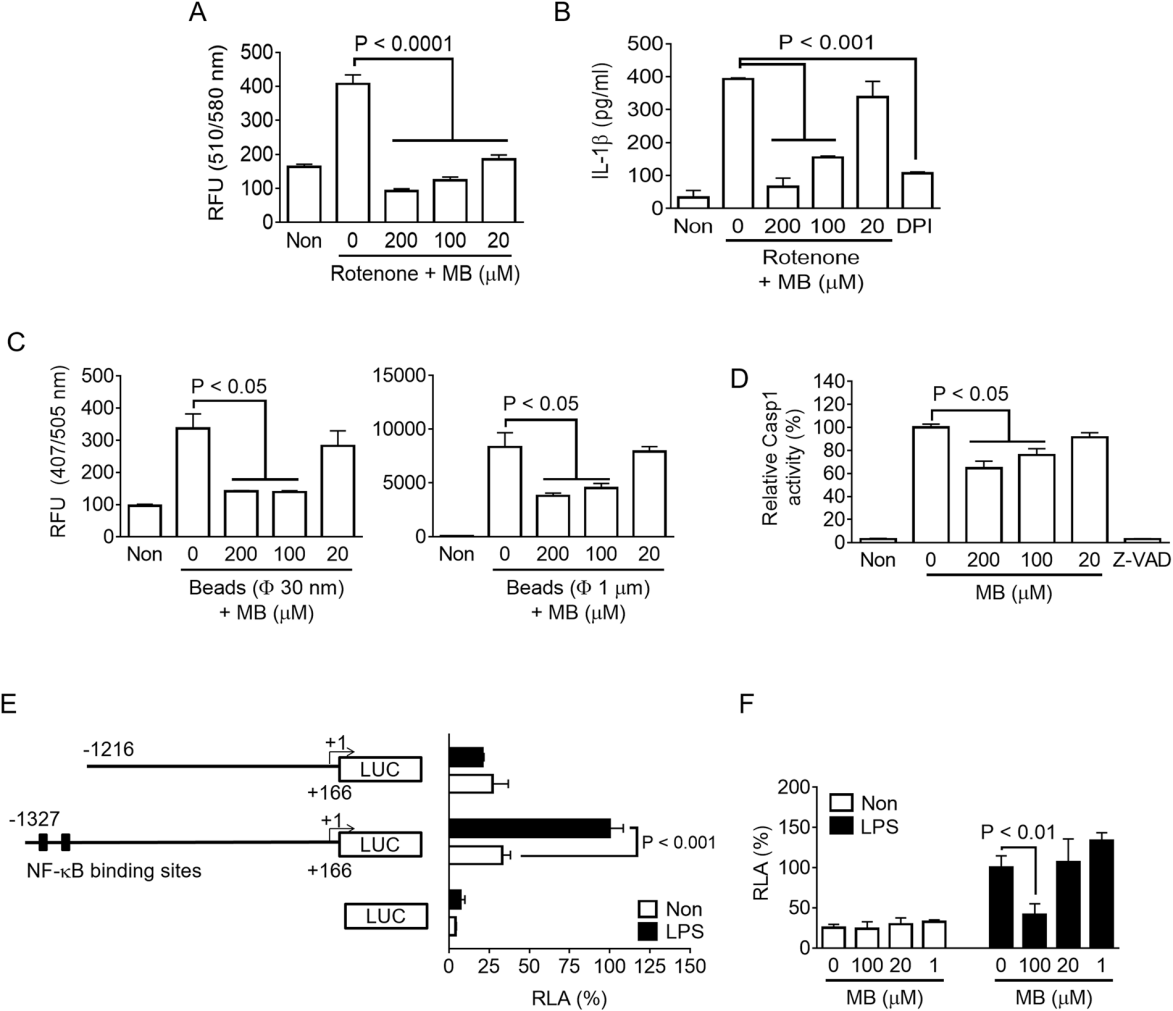
Effect of MB on mitochondrial ROS production, phagocytosis, caspase-1 activity, and NLRP3 promoter activity. (**A**) BMDMs were treated with rotenone (160 μM) and the indicated dosages of MB for 6 h, after which mitochondrial ROS generation was analyzed. (**B**) LPS-primed BMDMs were treated with rotenone with/without MB, and secretion of IL-1β was measured by ELISA. (**C**) BMDMs were incubated with two different diameters (30 nm or 1 μm) of fluorescent beads for 6 h, followed by measurement of intracellular beads based on florescence intensity. (**D**) Recombinant human caspase-1 (rhCasp1) was incubated with its substrate (YVAD-pNA) in the presence of MB as indicated. (**E**) Luciferase-expressing plasmids (pGL3) controlled by mouse NLRP3 promoters (−1,327 nt to + 166 nt or −1,216 nt to + 166 nt) were transfected into RAW 264.7 cells, which were treated with/without LPS. Left schematic figures indicate the mouse NLRP3 promoter regions (filled boxes, NF-κB binding sites; LUC, luciferase gene). (**F**) RAW 264.7 cells were transfected with pGL3 (−1,327 nt to + 166 nt) and treated with MB and/or LPS. Relative luciferase activity (RLA) was analyzed. Bar graph presents the mean ± SD.

Next, we investigated the molecular pathway responsible for the anti-NLRP3 inflammasome effect of MB. We first tested the effect of MB on mitochondrial reactive oxygen species (ROS) production, a well-characterized molecular mediator that triggers NLRP3 inflammasome assembly^14^. As shown in Fig. 3A, mitochondrial ROS levels in LPS-primed BMDMs were induced by rotenone treatment, which interrupts electron transport in mitochondria, and rotenone-mediated ROS production was attenuated by MB co-treatment in a dose-dependent manner. Rotenone treatment in LPS-primed BMDMs induced IL-1β secretion as expected (Fig. 3B). Rotenone-mediated IL-1β secretion was inhibited by MB similar to the effect of diphenyleneiodonium (DPI), which blocks cellular and mitochondrial ROS production. This result suggests that MB blocks mitochondrial ROS generation, resulting in NLRP3 inflammasome inhibition. Indeed, MB has been reported to ameliorate mitochondrial function as well as act as an antioxidant^15^.

We further assessed the effect of MB on phagocytosis since MB also inhibited MSU crystal- mediated NLRP3 inflammasome activation (Fig. 1F). In general, crystals must be eaten by macrophages to induce phagosome rupture, resulting in cytosolic cathepsin B release and NLRP3 inflammasome activation^16^. We treated with fluorescence-conjugated latex beads (30 nm or 1 μm) to elucidate the effect of MB on phagocytosis in LPS-primed BMDMs (Fig. 3C). In our results, MB attenuated phagocytosis, implying that MB directly blocks phagocytosis before attenuating NLRP3 inflammasome activation.

Furthermore, we determined the effect of MB on caspase-1 activity, which induces maturation of IL-1β/18. MB inhibited upstream events of inflammasome activation, such as Asc pyroptosome formation, phagocytosis, and the priming step. As shown in Fig. 3D, human recombinant caspase-1 was incubated with its substrates in the presence of MB or Z-VAD-FMK, a pan caspase inhibitor, in a tube. As the result, MB inhibited casaspase-1 activity in a dose-dependent manner. Based on a previous report^17^, we speculate that MB inhibits the activity of caspase-1 by oxidizing the catalytic cysteine.

To elucidate the molecular mechanism of MB-mediated inhibition on the priming step, we constructed two promoter activity assaying plasmids containing luciferase under the control of the mouse NLRP3 promoter (−1,327 nucleotides (nt) to + 166 nt or −1,216 nt to + 166 nt) based on a previous study^18^. As expected, the construct possessing two NF-κB-binding sites between −1,327 nt to −1,261 nt induced relative luciferase activity (RLA) in response to LPS treatment, whereas the other construct (-1,216 nt to + 166 nt) without NF-κB-binding sites did not exhibit altered RLA upon LPS treatment (Fig. 3E). We further compared RLA of the construct (−1,327 nt to + 166 nt) containing NF-κB-binding sites in the presence of MB (Fig. 3F). In the results, elevation of RLA by LPS treatment was attenuated by co-treatment with MB. This result implies that MB inhibits the priming step of inflammasome activation and cytokine expression via attenuation of NF-κB signaling.

### Methylene blue inhibits NLRC4 and AIM2 inflammasomes

**Figure 4:**
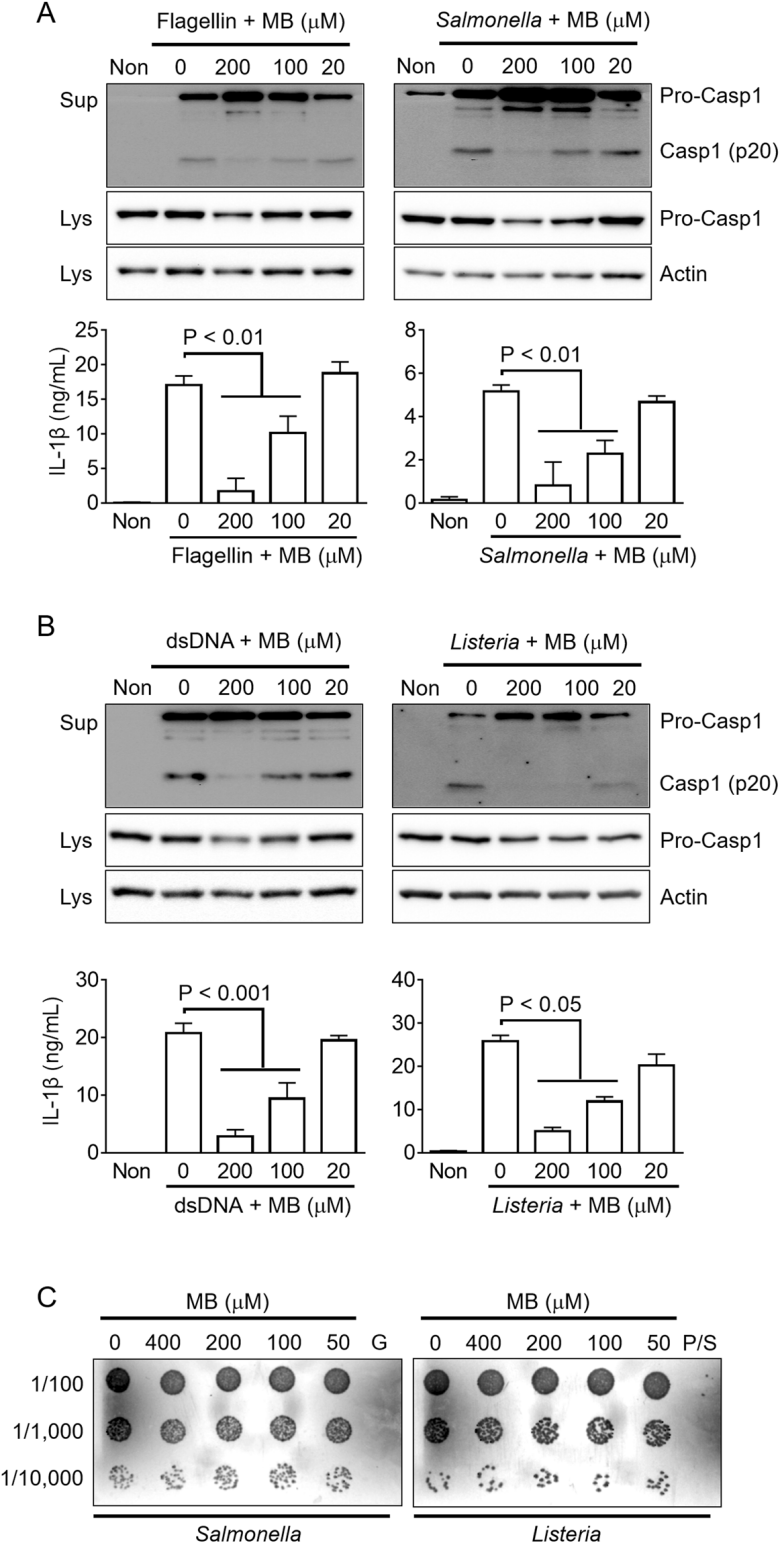
Effect of MB on NLRC4 or AIM2 inflammasome activation. (**A**) For NLRC3 inflammasome activation, LPS-primed BMDMs were treated with the indicated dosage of MB in the presence of flagellin (0.5 μg/mL) or *Salmonella* typhimurium (MOI 3.5) for 1 h. (**B**) For AIM2 inflammasome activation, macrophages were primed with LPS and treated with MB and dsDNA (1 μg/mL) for 1 h or *Listeria monocytogenes* (MOI 35) for 3 h. Secretions of caspase-1 (Casp1) and IL-1β were measured by immunoblotting and ELISA. (**C**) For bactericidal properties of MB, diluted *Salmonella* or *Listeria* were cultured in LB or BHI plates with the indicated concentration of MB or antibiotics. G gentamycin (50 μg/mL); P/S, penicillin (100 I.U.), and streptomycin (100 μg/mL). All immunoblot and bacterial growing data shown are representative of at least three independent experiments. Bar graph presents the mean ± SD.

We further elucidated the effect of MB on other inflammasomes such as NLRC4 and AIM2. To trigger NLRC4 inflammasome activation, LPS-primed BMDMs were transfected with flagellin or inoculated with *Salmonella* typhimurium (Fig. 4A). Similar to its effect on the NLRP3 inflammasome, MB dose-dependently inhibited flagellin- or *Salmonella*-mediated IL-1β and Caps1 secretion. In addition, dsDNA transfection and *Listeria monocytogenes* infection were utilized to activate AIM2 inflammasome in LPS-primed macrophages (Fig. 4B). dsDNA- and *Listeria*-mediated IL-1β and caspase-1 secretions were significantly blocked by MB co-treatment. We also investigated the potential bactericidal effect of MB. As shown in Fig. 4C, MB did not interrupt growth of *Salmonella* or *Listeria*. Thus, MB inhibits activation of the NLRC4 and AIM2 inflammasomes.

### Methylene blue inhibits non-canonical inflammasomes

**Figure 5:**
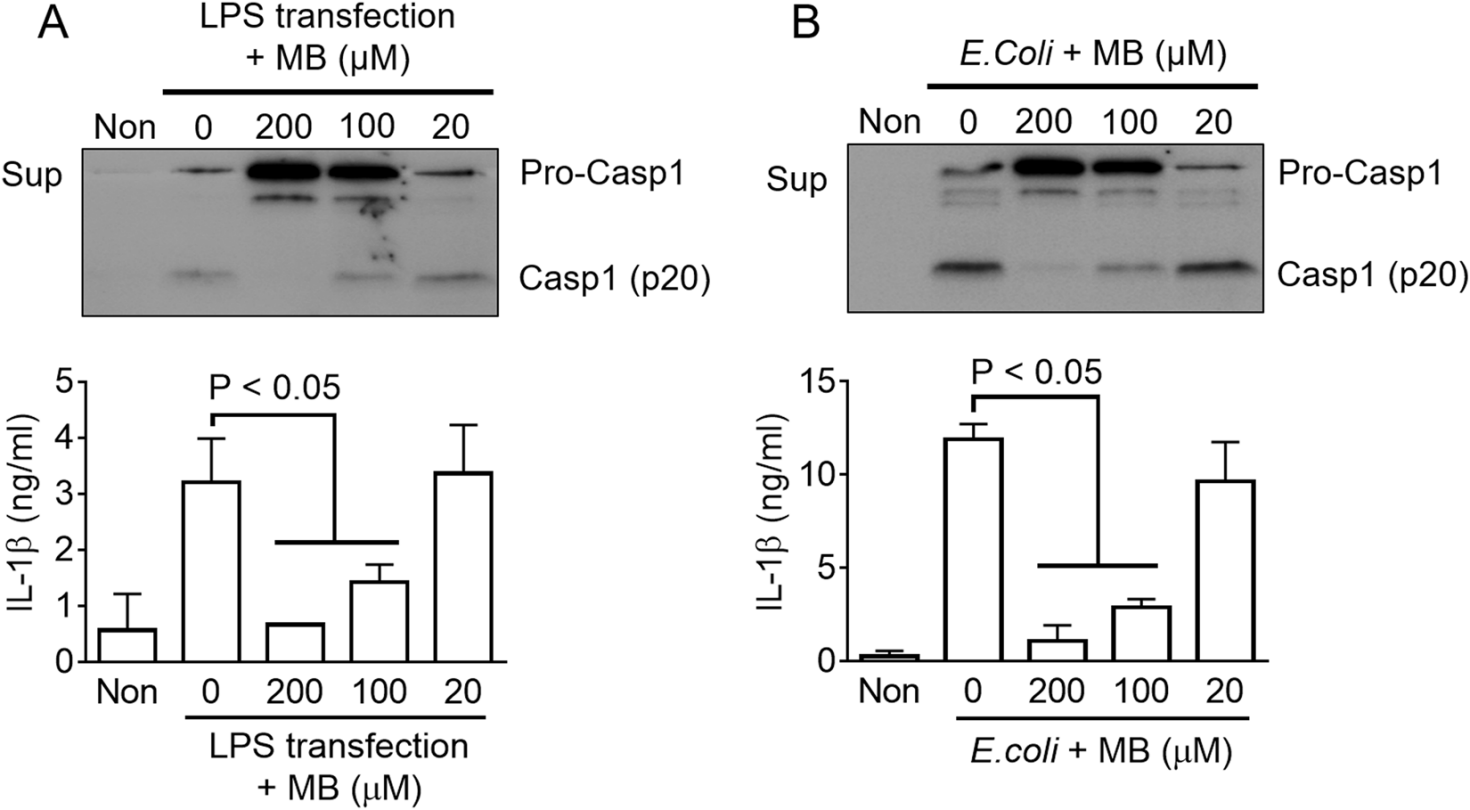
Effect of MB on non-canonical inflammasome activation. (**A**) LPS-primed BMDMs were transfected with LPS for non-canonical inflammasome activation in the presence of MB as indicated. (**B**) LPS-primed BMDMs were infected with *E.coli* (MOI = 10) in the presence of MB as indicated. Cspase-1 (Casp1) and IL-1β secretions were analyzed by immunoblotting and ELISA. All immunoblot data shown are representative of at least three independent experiments. Bar graph presents the mean ± SD.

We assessed whether or not MB attenuates activation of the non-canonical inflammasome, which acts as an upstream signal of NLRP3 inflammasome activation and is tightly involved in LPS-induced lethality^11,19^. For non-canonical inflammasome activation, LPS-primed BMDMs were transfected with LPS (Fig. 5D) or inoculated with *E.coli* (Fig. 5E). As the result, MB attenuated caspase-1 and IL-1β secretion resulting from LPS or *E.coli*-mediated non-canonical inflammasome activation. This result implies that MB not only inhibits the canonical inflammasome but also blocks the non-canonical inflammasome.

### Methylene blue inhibits inflammasomes and cytokine expression in a human cell line, THP-1

**Figure 6:**
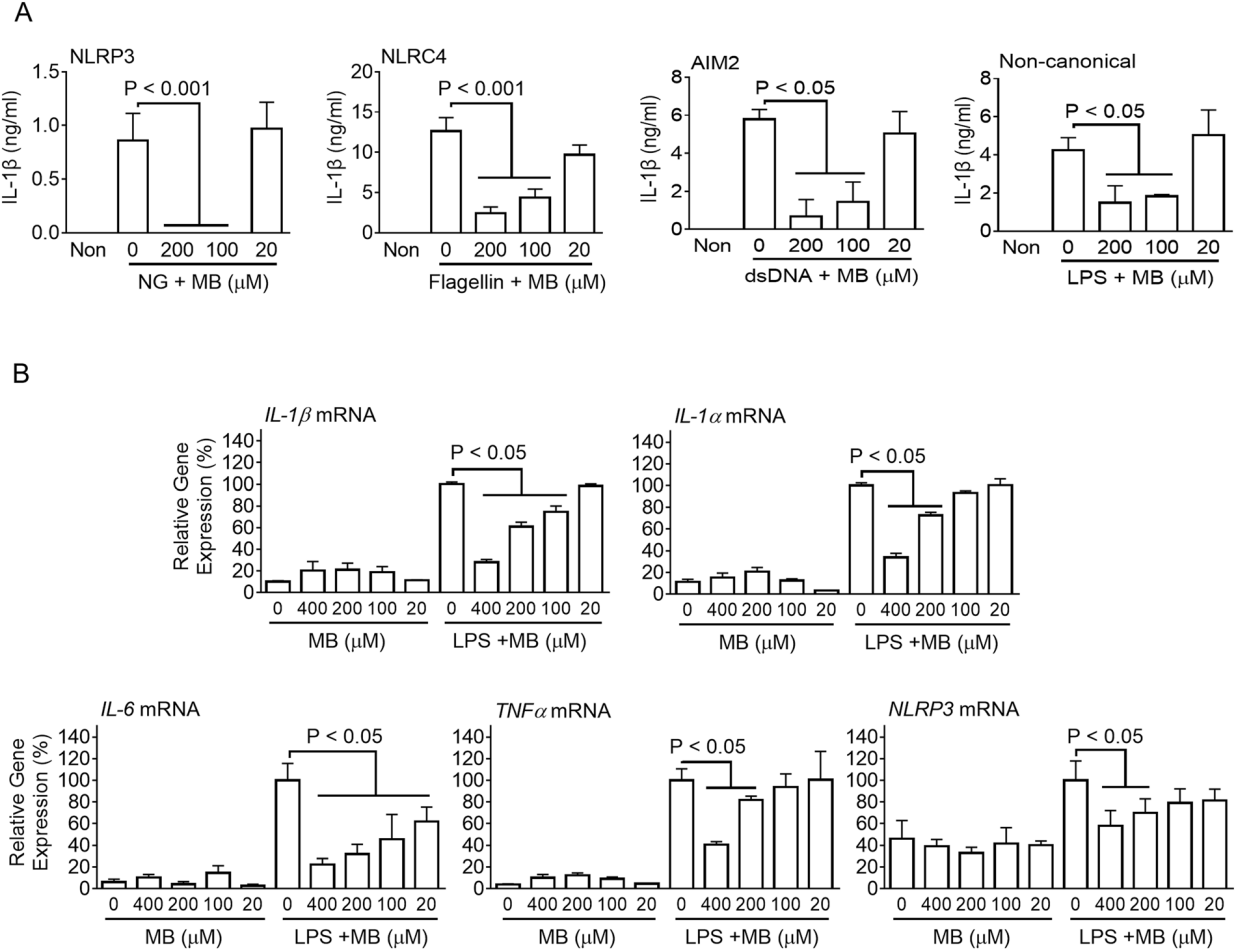
Effect of MB on inflammasome activation in a human monocyte-like cell line, THP-1. (**A**) PMA-treated THP-1 cells were primed with LPS and then subjected to NG treatment and transfection of flagellin, dsDNA, and LPS in order to activate NLRP3, NLRC4, AIM2, and non-canonical inflammasomes. Secretion of IL-1β was analyzed by ELISA. (**B**) Expression levels of human *IL-1β, IL-1α, IL-6, TNFα*, and *NLRP3* mRNAs were quantitated by real-time PCR. Bar graph presents the mean ± SD.

Although MB presented anti-inflammasome properties in murine macrophages, we further investigated whether or not MB acts similarly in human cells. We adopted a human monocyte-like cell line, THP-1, and elucidated the effect of MB on inflammasome activation. THP-1 was differentiated by phorbol 12-myristate 13-acetate (PMA) treatment and subjected to priming with LPS. LPS-primed THP-1 cells showed activation of NLRP3, NLRC4, AIM2, and non-canonical inflammasomes after NG treatment, flagellin transfection, dsDNA transfection, and LPS transfection with/without MB (Fig. 6A). In the results, MB dose-dependently attenuated IL-1β secretion resulting from NLRP3, NLRC4, AIM2, and non-canonical inflammasome activation, similar to mouse BMDMs. In addition, THP-1 cells were treated with MB with/without LPS, and gene expression of cytokines was analyzed by RT-PCR (Supplementary Fig. 2B) and real-time PCR (Fig. 6B). Up-regulation of *IL-1β, IL-1α, IL-6, TNFα*, and *NLRP3* mRNAs in response to LPS treatment was attenuated by MB co-treatment. Thus, MB attenuated the priming and activation steps of inflammasome activation in a human cell line, THP-1, similar to murine macrophages.

### Methylene blue ameliorates inflammasome-mediated diseases in mice

**Figure 7:**
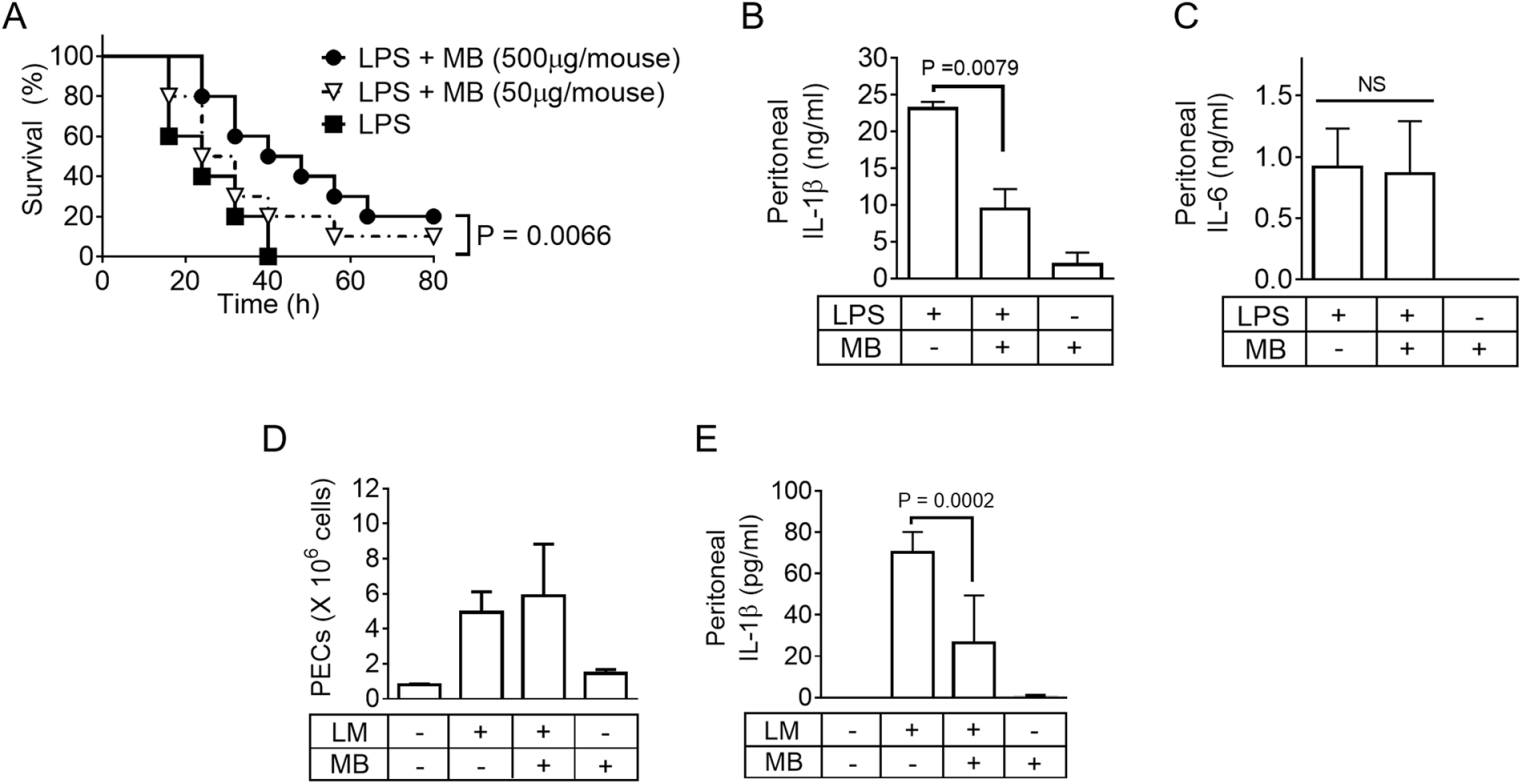
Effect of MB on inflammasome model animals and non-canonical inflammasome. (**A**) Mice (n = 10 per group) were intraperitoneally (ip) injected with LPS (25 mg/Kg) and/or MB as indicated. Survival rates were observed at the indicated times. (**B** and **C**), Mice (n = 5 per group) were ip-injected with LPS (4 mg/kg) and/or MB (500 μg/mouse). IL-1β (**B**) and IL-6 (**C**) secretion levels in peritoneal lavage fluids were measured. (**D** and **E**), Mice (n = 9 per group) were ip injected with *Listeria monocytogenes* (1,000 cfu in 200 μL of saline) and/or MB (500 μg/mouse). Number of peritoneal exudate cells (PECs, **B**) was calculated, and IL-1β (**C**) secretion levels of peritoneal lavage fluids were measured. Bar graph presents the mean ± SD.

We adopted two inflammasome-mediated disease models, LPS-induced lethality and *Listeria* peritonitis, to assess the anti-inflammasome properties of MB in mice. LPS-induced lethality, also called endotoxemic shock, is a well-defined animal model for the NLRP3 inflammasome^20^ and/or non-canonical inflammasome^11^. As shown in Fig. 7A, mice injected with LPS alone died within 4 h, but additional injection of MB to LPS-treated mice resulted in an increased survival rate (P = 0.0066, Log-rank test) in a dose-dependent manner. In addition, mice were injected with LPS and/or MB, after which peritoneal fluids were collected to measure IL-1β and IL-6 secretion after 6 h of injection. In the results, LPS injection induced both IL-1β and IL-6 secretion while MB co-treatment only attenuated LPS-mediated IL-1β secretion but not IL-6 secretion (Fig. 7B and C). This result implies that MB selectively inhibits inflammasome activation in animals, although MB blocked both cytokine production and maturation in cells. Next, mice were treated with *Listeria*, an AIM2 inflammasome trigger, with/without MB, after which the number of peritoneal exudate cells (PECs) and secretion of peritoneal IL-1β were determined. Although we investigated *Listeria*-induced lethality, our *Listeria* did not induce any lethality within 80 h. As shown in Fig. 7D, induction of PECs by *Listeria* injection was not altered by MB, implying that MB could not alter the chemotactic state. *Listeria* also induced peritoneal IL-1β secretion, which was attenuated by MB co-treatment (Fig. 7E). Thus, *in vivo* treatment with MB ameliorates LPS lethality through inhibition of NLRP3 and/or non-canonical inflammasome activation as well as *Listeria*-induced IL-1β production via inhibition of AIM2 inflammasome activation.

## Discussion

In this study, we assessed the effect of methylene blue (MB) on canonical (NLRP3, NLRC4, and AIM2) and non-canonical inflammasome activation. We demonstrated that MB acts as an anti-inflammasome agent. Specifically, MB attenuated specific inflammasome trigger-mediated IL-1β/18 and caspase-1 secretion as well as Asc pyroptosome formation. MB also blocked mitochondrial ROS production, which triggers NLRP3 inflammasome activation, as well as NLRP3 and pro-IL-1β expression, which are essential components for inflammasome activation. In addition, MB attenuated activity of casaspe-1, which directly induces maturation of IL-1β/18. The anti-inflammasome properties of MB were further confirmed in an animal model. MB treatment reduced LPS-induced lethality and *Listeria*-mediated IL-1β secretion. Taken together, we suggest that MB can inhibit both the beginning and end of canonical and non-canonical inflammasome activation.

MB has long been used for its therapeutic properties, and it is considered to be an effective agent to cure septic shock^21^. Sepsis models using rat, rabbit, and dog have suggested that MB infusion during endotoxic shock consistently increases mean arterial pressure (MAP) and peripheral vascular resistance while reducing catecholamine requirements in patients^22,23^. Vasodilation is mediated by cyclic guanosine monophosphate (cGMP) produced by soluble guanylate cyclases (sGC), and nitric oxide (NO) directly activates sGC^22^. As a mechanism of MB in septic shock, it has been suggested that MB down-regulates inducible nitric oxide synthase (iNOS) since LPS induces NO production, resulting in hypotension during septic shock^22^. In the present study, MB treatment increased the survival rates of LPS-treated mice (Fig. 4A). Although we demonstrated the inhibitory effect of MB on NLRP3 and/or non-canonical inflammasome activation for reduction of LPS lethality, blockage of NO production by MB might support increased survival rates in septic mice. On the other hand, we hypothesized that NO increases IL-1β secretion via inflammasome activation. For this, we treated L-arginine, an endogenous NO precursor, to LPS-primed BMDMs and assessed IL-1β secretion. As the result, L-arginine did not induce any IL-1β secretion in LPS-primed BMDMs (Supplementary Fig. 3). Thus, we conclude that MB attenuates inflammasome activation independent of NO production.

Although MB is a well-known antioxidant agent, the effect of MB on cytokine production in macrophages has been poorly studied. Based on the literature, MB treatment was shown to reduce *IL-1β* and increase *IL-10* gene expression in a LPS-treated microglia cell line, and MB infusion after traumatic brain injury in mice reduced expression of inflammatory genes in the hippocampus^3^. In addition, MB has been shown to attenuate expression of iNOS in response to LPS by inhibiting the binding affinity of transcription factors (NF-κB and STAT1) on the promoter region of *iNOS* gene^21^. In the present study (Fig. 2D), we demonstrated that MB treatment blocked the priming step of inflammasome activation as well as up-regulation of pro-IL-1β and NLRP3 proteins^12,13^. Both genes are tightly regulated by NF-κB signaling^12,13,18^, and MB might interrupt the binding of NF-κB to the promoter regions of *pro-IL-1β* and *NLRP3* genes, as shown in previous literature^21^.

MB is an oxidation-reduction (redox) agent previously used safely in humans as an antidote for certain metabolic poisons^1^. In addition, MB prevents the formation of superoxide and nitric oxide in mitochondria and is able to improve brain oxidative metabolism by enhancing mitochondrial oxygen consumption^1^. In animal studies, MB counteracts the damaging effect of rotenone, an inhibitor of the mitochondrial electron transfer complex I, on retinal neurons^24^. Thus, MB is suggested as a potential therapeutic target for mitochondrial dysfunction. Mitochondrial dysfunction plays a determinant role in a number of acute and chronic inflammatory diseases^25^. Mitochondrial dysfunction acts upstream of NLRP3 activation by providing ROS to trigger NLRP3 oligomerization or by inducing α-tubulin acetylation to relocate mitochondria in proximity to NLRP3^14,26^. Based on our results and previous reports, we conclude that MB attenuates inflammasome activation by improving mitochondrial function.

MB is used in several diagnostic procedures as a staining agent, including bacteria staining and intraoperative tissue staining^27^. For example, MB and epinephrine-containing saline are injected into the submucosal layer around a polyp during endoscopic polypectomy. MB helps to identify submucosal tissue after the polyp is removed, which is useful for determining whether more tissue should be removed or if there is a high risk for perforation^28^. MB is also used as a dye in chromoendoscopy and is sprayed onto the mucosa of the gastrointestinal tract to identify dysplasia or pre-cancerous lesions^29^. In surgeries such as sentinel lymph node dissections, MB can be used to visually track lymphatic drainage of involved tissues^30^. Likewise, MB is added to bone cement during orthopedic surgery to easily distinguish cement from native bone^31^. Thus, MB is used conservatively during the above procedures as a staining and/or coloring agent. However, we suggest that MB has an additional effect than just a staining molecule based on its ability to attenuate inflammasome activation and cytokine expression. Thus, the anti-inflammatory properties of MB may prevent unwanted complications after surgical procedures.

Inflammasome dysregulation has been implicated in neurologic disorders and metabolic diseases, neither of which are traditionally considered to be inflammatory diseases but which are increasingly recognized as having an inflammatory component that significantly contributes to the disease process and drives many forms of cancer in humans^5^. Therefore, researchers have become interested in the regulation of inflammasome activation. So far, several reagents such as recombinant IL-1 receptor antagonist (anakinra), neutralizing IL-1β antibody (canakinumab), soluble decoy IL-1 receptor (rilonacept), IL-18–binding protein, soluble IL-18 receptors, and anti–IL-18 receptor monoclonal antibodies have been developed and applied to control inflammasome-mediated diseases^5^. These reagents only control events downstream of inflammasome activation such as blockage of IL-1β/-18 signaling. However, we have attempted to screen natural compounds that selectively control events upstream of inflammasome activation^32–38^. Based on our finding, MB has the most wide range of anti-inflammasome agents and controls several events upstream of inflammasome activation. Specifically, MB blocks the NLRP3, NLRC4, and AIM2 inflammasomes as well as non-canonical inflammasome. In addition, MB attenuates crystal phagocytosis, the priming step of inflammasome activation, Asc speck formation, and caspse-1 activation.

## Materials and Methods

### Cell culture

Bone marrow-derived macrophages (BMDMs) were cultured as described in detail elsewhere^38,39^. In brief, femur and tibia bones from C57BL/6 mice (6-12-weeks-old; Narabio Co., Seoul, Republic of Korea) were collected, and bone marrow cells from all bones were flushed out. Cells were than cultured in Dulbecco’s Modified Eagle Medium (DMEM; WELGENE Inc. Daegu, Republic of Korea or Capricorn Scientific GmbH, Ebsdorfergrund, Germany) supplemented with 10% fetal bovine serum (FBS; Corning cellgro, Manassas, VA, USA or Capricorn Scientific GmbH) and 1% penicillin and streptomycin solution (P/S; Corning cellgro, 10,000 I.U. of penicillin and 10,000 µg/mL of streptomycin) in L-929 cell-conditioned medium containing granulocyte/macrophage colony-stimulating factor. Cells were plated in non-tissue culture-treated Petri dishes (SPL Life Science Co., Phcheon-si, Gyeonggi-do, Republic of Korea) and incubated at 37 °C in 5% CO_2_ atmosphere for 7 days.

THP-1 or RAW 264.7 cells were obtained from the Korean Cell Line Bank (KCLB No. 40202 or 40071; Seoul, Republic of Korea) and maintained in RPMI 1640 medium (WELGENE Inc. or Capricorn Scientific GmbH) or DMEM containing 10% FBS and P/S at 37 °C in a 5% CO_2_ atmosphere. THP-1 cells were differentiated into macrophage-like cells using PMA (100 nM; Cat. tlrl-pma, InvivoGen) for 24 h.

### Cell treatment

For inflammasome activation, BMDMs (1.0 × 10^6^ cells per well) or PMA-treated THP-1 (1.0 × 10^6^ cells per well) in RPMI 1640 containing 10% FBS and P/S were seeded in 12-well tissue culture plates (SPL Life Science Co.) and primed with lipopolysaccharide (LPS, 1 μg/mL; L4130, Sigma-Aldrich Co., St. Louis, MO, USA) for 3 h according to a previous study^32^. LPS-primed BMDMs or THP-1 were given fresh media (RPMI 1640, 350 μL/well in 12-well plates) without FBS or antibiotics in the presence of nigericin (40 μM, NG; 4312 Tocris Bioscience, Bristol, UK) for 1 h, monosodium uric acid (400 μg/mL, MSU, U2875, Sigma-Aldrich Co.) for 6 h, flagellin (0.5 μg/mL; tlrl-stfla, InvivoGen, San Diego, CA, USA) with Lipofectamine 2000 (10 μL/mL, Invitrogen, Grand Island, NY, USA) for 1 h, *Salmonella* typhimurium (multiplicity of infection [MOI] 3.5) for 1 h, double-stranded DNA (1 μg/mL, dsDNA) with jetPRIME^TM^ (2 μL/mL, Polyplus-transfection Inc., Illkirch, France) for 1 h, rotenone (160 μM; sc-203242, Santa Cruz Biotechnology) for 6 h, *Listeria monocytogenes* (MOI 35) for 3 h, LPS (15 μg/mL, Sigma-Aldrich Co) with Lipofectamine 2000 (10 μL/mL, Invitrogen) for 6 h, or *Escherichia coli* (MOI 10, DH5α, Invitrogen) with methylene blue (0, 20, 100, or 200 μM, MB, 2278-4125, Daejung Chemicals & Materials Co., Gyeonggi-do, Republic of Korea or A5105, Tokyo Chemical Industry Co., LTD. Tokyo, Japan) or diphenyleneiodonium (DPI, 0504, 200 μM, Tocris Bioscience). Schematic diagram for inflammasome activation is shown in the bottom of Fig. 1C.

To determine the effect of MB on the priming step, BMDMs or THP-1 were treated with MB (0, 20, 100, or 200 μM) with/without LPS (10 ng/mL) for 30 min, after which cells were given fresh media with/without LPS (10 ng/mL) for 2.5 h. Details of the treatment are presented in the bottom of Fig. 2A.

### Western blotting sample preparation

After inflammasome activation, cellular supernatant (Sup; 350 μL of RPMI 1640) was transferred into a new tube, and remaining BMDMs were lysed with 100 μL of mild lysis buffer (150 mM NaCl, 1% Triton X-100, 50 mM Tri-base, pH 8.0) containing proteinase inhibitor cocktail (#M250-1, AMRESCO LLC, Solon, OH, USA)^36,37^. The lysate (Lys) was transferred into a new tube and collected by centrifugation at 15,000 rcf for 5 min. The remaining pellet was washed two times with PBS and then re-suspended and cross-linked with 2 mM suberic acid bis (Sigma-Aldrich Co.) for 1 h, followed by centrifugation at 15,000 rcf for 5 min. The cross-linked pellets (Pellet) were re-suspended in 50 μL of 2 X loading dye buffer (116 mM Tris, 3.4% SDS, 12% glycerol, 200 mM DTT, 0.003% bromo phenol blue)^35^. Sup, Lys, and Pellet were subjected to Western blot assay.

### Western blot analysis

Unless otherwise indicated, all materials for Western blot analysis were purchased from BIO-RAD (Hercules, CA, USA). Sup and Lys were separated by SDS-PAGE (10% or 16%) using running buffer and transferred onto a polyvinylidene difluoride membrane (PVDF; 10849 A, Pall Co., Port Washington, NY, USA) using transfer buffer. The membranes were blocked with 3% skim milk and probed overnight at 4 °C with anti-mouse IL-1β antibody (AF-401-NA, R&D Systems, Minneapolis, MN, USA), anti-caspase-1 antibody (AG-20B-0042, AdipoGen Co., San Diego, CA, USA), anti-NLRP3 antibody (AG-20B-0014-C100, AddipoGen Co.), anti-Asc antibody (sc-22514, Santa Cruz Biotechnology, Santa Cruz, CA, USA), or anti-actin antibody (sc-1615, Santa Cruz Biotechnology). Membranes were further probed with HRP-conjugated 2^nd^ anti-sera (sc-2020, sc-2005 or sc-2004, Santa Cruz Biotechnology) and visualized by Power-Opti ECL^TM^ solution (BioNote Co., Gyeonggi-do, Republic of Korea) and a Cooled CCD camera System (AE-9105 EZ-Capture II, ATTO Technology, Tokyo, Japan).

### Reverse transcription polymerase chain reaction (RT-PCR)

Total RNA was extracted using NucleoZOL (MACHEREY-NAGEL GmbH & Co. KG, Postfach, Düren, Germany) and reverse-transcribed into first-strand complementary DNA (cDNA) using an M-MLV cDNA Synthesis kit (Enzynomics, Daejeon, Korea). Transcription was amplified using a SimpliAmp^TM^ Thermal Cycler (Thermo Fisher Scientific Inc. Grand Island, NY, USA) and nTaq polymerase (Enzynomics). PCR products were visualized by agarose gel electrophoresis, ethidium bromide staining, and EZ-Capture^TM^ II (ATTO Technology). In addition, levels of gene expression were quantified using SYBR Green (TOPreal^TM^ qPCR 2X PreMIX, Enzynomics) and an Eco Real-Time PCR system (Illumina, San Diego, CA, USA). Quantitation was normalized with β-actin (*Actb*). Information on gene-specific primers is listed in Supplementary Table 1.

### Cytotoxicity assay

BMDMs (1 × 10^6^ cells per well) were plated in a 6-well plate (SPL Life Science Co.) and treated with MB (0, 100, or 200 μM; Daejung Chemicals & Materials Co.). After 3 h, cells were lifted by a scraper in cold PBS and analyzed using an automated cell counter (Moxi Z^TM^, ORFLO Tech., ID, USA).

### Mitochondrial reactive oxygen species assay

BMDMs (1.25 × 10^5^ cells per well) plated in a 96-well black plate (SPL Life Science Co.) were incubated with MitoSOX^TM^ Red mitochondrial superoxide indicator (2.5 μM, M36008, Invitrogen) for 30 min at 37 °C^34^. Cells were treated with rotenone (160 μM; sc-203242, Santa Cruz Biotechnology) in the presence of MB (0, 20, 100, or 200 μM) for 6 h at 37 °C. The plates were readout using a plate reader (510/580 nm, Synergy™ H1 Hybrid Multi-Mode Reader, BioTek, Winooski, VT, USA).

### Phagocytosis assay

For the analysis of phagocytosis, BMDMs (1 × 10^4^ cells per well) plated in a 96-well black plate (SPL Life Science Co.) were incubated with 0.005% fluorescent carboxyl-modified polystyrene latex beads (30 nm [L5155] or 1 μm [L4655], Sigma-Aldrich Co.) in the presence of MB (0, 20, 100, or 200 μM) for 6 h. After washing three times with PBS, fluorescence intensity was measured using a plate reader (470/505 nm, Synergy™ H1 Hybrid Multi-Mode Reader, BioTek).

### NLRP3 promoter study

Unless otherwise indicated, all restriction enzymes and cloning enzymes were obtained from Enzynomics. Two promoter regions (−1,327 nt to + 166 nt or −1,216 nt to + 166 nt) of the mouse NLRP3 gene were amplified by PCR using Ex Taq^®^ (TaKaRa Bio Inc., Seoul, Korea), and specific primers are listed in Supplementary Table 1. The two PCR products were cloned with TA cloning vector (TOPcloner^TM^ TA core Kit) and then sub-cloned into pGL3-Basic (Promega Corporation, Madison, WI, USA) by SacI and XhoI digestion. Raw 264.7 cells (2 × 10^6^ cells) were plated in 24-well plates (SPL Lifesciences) 1 day before transfection and then transfected with the two promoter constructs (150 ng / well) and Renilla TS (5 ng / well, Promega) using jetPRIME^TM^. After incubation overnight, cells were treated with the indicated dosages of MB and/or LPS (10 ng/ml) for 30 min and then replaced with fresh media with/without LPS (10 ng/ml) similar to the bottom of Fig. 2A. After 2.5 h, cellular lysates were assayed for luciferase activity using the Dual-Luciferase Assay System (Promega) and a Synergy™ H1 Hybrid Multi-Mode Reader, BioTek). Relative luciferase activity (RLA) was calculated as luciferase activity / renilla luciferase activity.

### Bacterial growth

*Salmonella* typhimurium^33^, *Listeria monocytogenes* (Korean Culture Center of Microorganisms, Seoul, Republic of Korea), and *Escherichia coli* (*E.coli*, DH5α, Invitrogen) were grown in Luria-Bertani (LB, Laboratories Conda, Madrid, Spain) broth for *Salmonella* and *E.coli* or Brain Heart Infusion (BHI, Laboratories Conda) broth for *Listeria*. Bacteria were growth for 18 h with shaking at 37 °C. *Salmonella* grown in media (2%) were transferred to fresh LB broth and further incubated for 3 to 4 h with shaking at 37 °C. To elucidate the effect of MB on bacterial growth, *Salmonella* and *Listeria* were grown in broth containing PBS, MB, gentamycin (50 μg/mL, Komipharm International Co., Ltd., Gyeonggi-do, Republic of Korea) or P/S (100 I.U. of penicillin and 100 µg/mL of streptomycin) for 1 h at 37 °C, followed by the spot-titer plating method on a LB or BHI plate for 18 h at 37 °C.

### Animal experiments

Male C57BL/6 mice (8 to 10-weeks-old) were purchased from Narabio Co. (Seoul, Republic of Korea). All mice were maintained under a 12 h light/dark cycle at 24 °C. Animals were provided standard sterile food and water *ad libitum*, after which they were allowed to adjust to the environment for 1 week. For LPS lethality, mice (5 mice/group/trial, total n = 10 per group) were intraperitoneally (ip) injected with MB (2 or 20 mg/kg, Daejung Chemicals & Materials Co.) after 1 h of LPS (25 mg/kg, Sigma-Aldrich Co.) or saline (200 μL) ip treatment^40^. Mouse mortality was observed every 8 h for 4 days. For peritoneal cytokine secretion, mice (3 mice/group/trial, total n = 9 per group) were intraperitoneally (ip) injected MB (20 mg/kg) after 30 min of *Listeria* (1,000 cfu per mouse) ip treatment. In addition, mice (five mice/group) were ip-injected with MB (20 mg/kg) after 30 min of LPS (4 mg/kg) ip treatment. After 5.5 h, mice were sacrificed via CO_2_ exposure. Peritoneal cavities were washed with 5 mL of PBS, and peritoneal exudate cells (PECs) were analyzed by a cell counter (Moxi Z^TM^, ORFLO Technologies). Lavage fluids were collected for further analysis. All animal experiments were carried out in accordance with the National Institutes of Health Guide for the Care and Use of Laboratory Animals and approved by the Institutional Animal Care and Use Committee of Kangwon National University (IACUC; approval no. KW-170110-1).

### Cytokine detection using ELISA

To quantitate secreted cytokines, cell culture supernatants of BMDMs or peritoneal lavage fluids were measured by ELISA Kits for human IL-1β (DY201-05, R&D Systems), mouse IL-1β (DY401, R&D Systems), mouse IL-6 (M6000B, R&D Systems), and mouse IL-18 (BMS618/3, eBioscience, San Diego, CA, USA). The ELISA plates were readout using a Synergy™ H1 Hybrid Multi-Mode Reader, BioTek).

### Caspase-1 activity assay

Caspase-1 activity was measured using a Caspase-1/ICE Fluorometric Assay Kit (K110, BioVison Inc.) according to the manufacturer’s protocol. Briefly, human recombinant caspase-1 (1 unit/rx, 1081, BioVison Inc., CA. USA) was incubated with YVAD-pNA, a substrate of caspase-1, in the presence of MB or Z-VAD-FMK (a pan caspase inhibitor, FMK001, R&D Systems).

### Statistical analyses

Statistical analyses were performed using a one-way ANOVA (Turkey’s multiple comparisons test) for multiple groups and log-rank (Mantel-Cox) test for lethality measurement using GraphPad Prism 6 (GraphPad Software, San Diego CA). P value is indicated in the figure.

## Electronic supplementary material

Supplementary
