# Supplementary material for: Methylene blue inhibits NLRP3, NLRC4, AIM2, and non-canonical inflammasome activation
Source: Sci Rep. 2017 Sep 29;7:12409. doi: 10.1038/s41598-017-12635-6 (PMC5622101; doi:10.1038/s41598-017-12635-6)
Supplement: Supplementary file 1 — Supplementary [file 41598_2017_12635_MOESM1_ESM.doc]

Supplementary Information

**Methylene blue inhibits NLRP3, NLRC4, AIM2, and non-canonical inflammasome activation**

Huijeong Ahn^1^, Seung Goo Kang^2^, Sung-il Yoon^3^, Hyun-Jeong Ko^4^,

Pyeung-Hyeun Kim^2^, Eui-Ju Hong^5^, Beum-Soo An^6^, Eunsong Lee^1^, and Geun-Shik Lee^1#^

^1^College of Veterinary Medicine and Institute of Veterinary Science; ^2^Department of Molecular Bioscience, School of Biomedical Science; ^3^Division of Biomedical Convergence, College of Biomedical Science; ^4^Laboratory of Microbiology and Immunology, College of Pharmacy, Kangwon National University, Chuncheon, Gangwon, 24341, Republic of Korea.

^5^College of Veterinary Medicine and Institute of Veterinary Science, Chungnam National University, Daejeon, Republic of Korea.

^6^Department of Biomaterial Science, College of Natural Resources and Life Science, Pusan National University, Gyeongsangnam-do, Republic of Korea

**Supplementary Figure 1**.


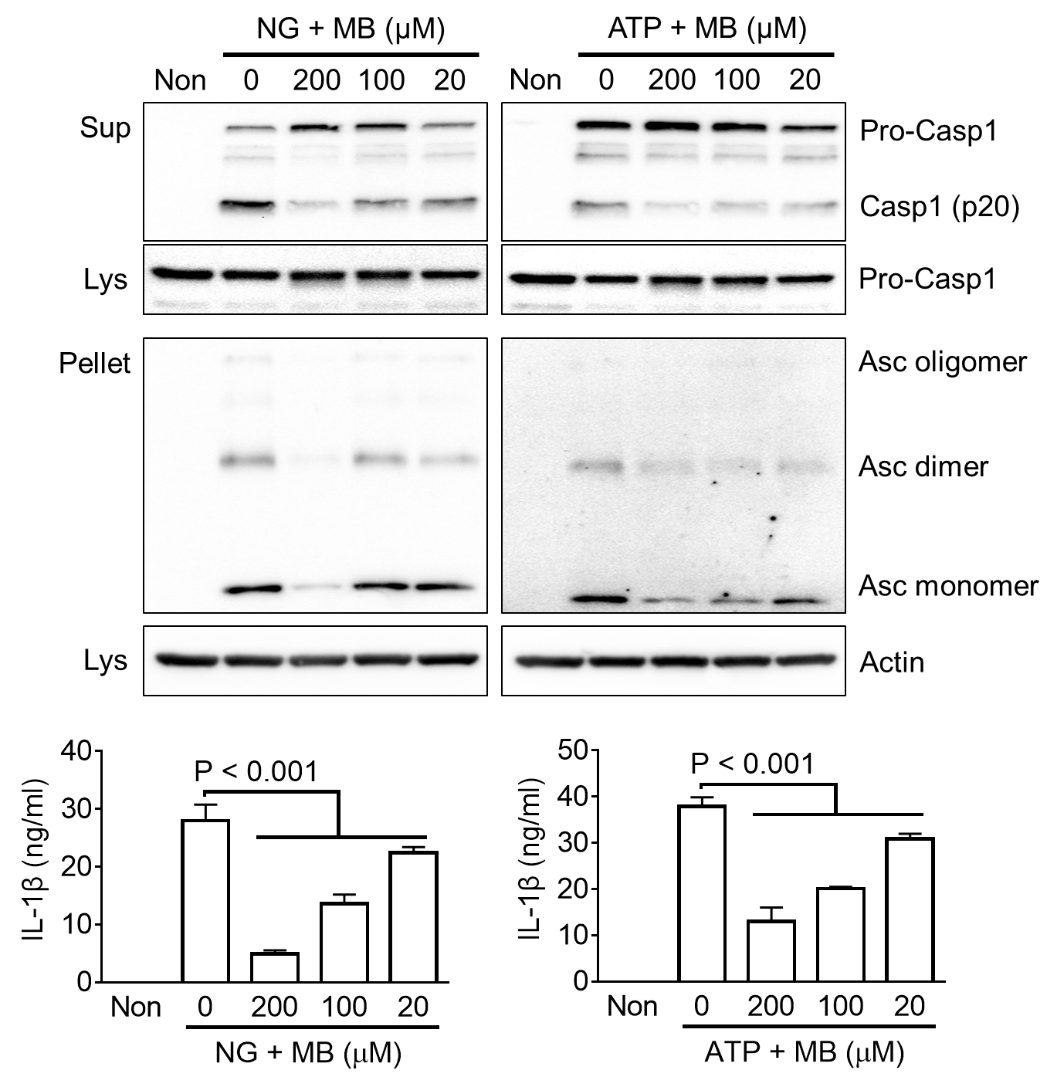


**Supplementary figure 1**. Effects of NG and ATP on Casp1 and IL-1β secretion and Asc speck formation

LPS-primed BMDMs were treated with NG or ATP and increasing dosages of MB as indicated. Casp1 secretion and Asc speck formation were detected by immunoblotting, and IL-1β secretion was measured by ELISA.

**Supplementary Figure 2**.


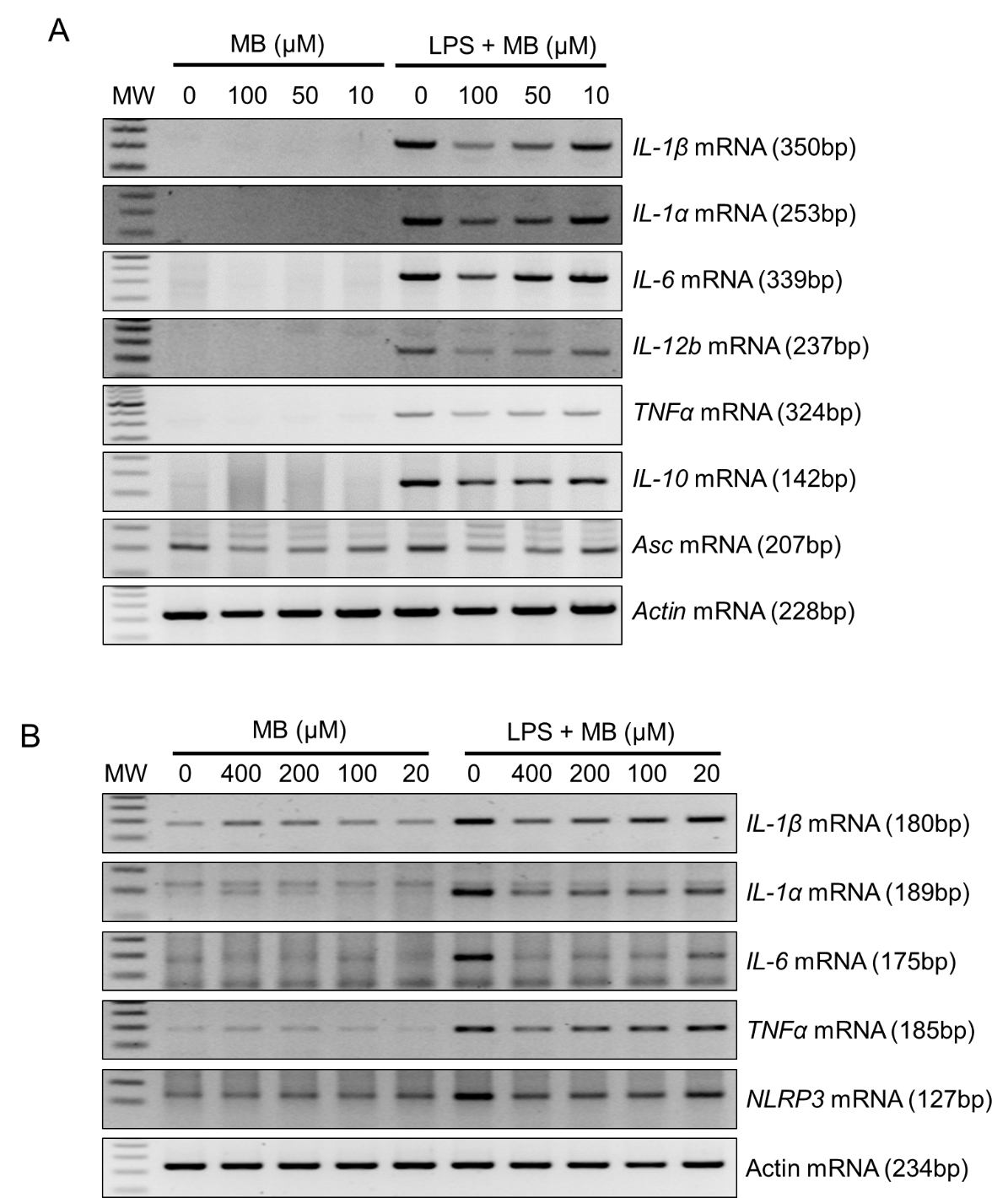


**Supplementary figure 2**. Effect of MB on expression of cytokines

**A**, Mouse BMDMs were treated with the indicated concentration of MB with/without LPS (10 ng/mL). **B**, THP-1, human monocyte-like cell line, was treated with the indicated concentration of MB with/without LPS (10 ng/mL). Gene expression levels were analyzed by RT-PCR. MW, molecular weight (100 bp ladder).

**Supplementary Figure 3**.


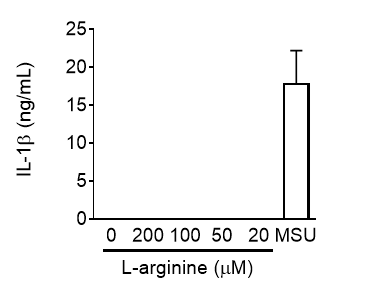


**Supplemental Figure 1**. Effect of L-Arginine on IL-1β secretion

LPS-primed BMDMs were treated with L-arginine, an endogenous nitric oxide precursor, as indicated. IL-1β secretion was measured by ELISA.

**Supplementary Table 1.** Primer sequences

| Gene Name | Species | GeneBank ID | Direction | Sequence (5' to 3') | Expected band size (bp) |
| --- | --- | --- | --- | --- | --- |
| *IL-1β* | Mouse | NM_008361 | F | CAG GCA GGC AGT ATC ACT CA | 350 |
|  |  |  | R | AGG CCA CAG GTA TTT TGT CG |  |
| *IL-6* | Mouse | NM_031168 | F | GTT CTC TGG GAA ATC GTG GA | 339 |
|  |  |  | R | GGA AAT TGG GGT AGG AAG GA |  |
| *TNFα* | Mouse | NM_013693 | F | ACG GCA TGG ATC TCA AAG AC | 324 |
|  |  |  | R | CGG ACT CCG CAA AGT CTA AG |  |
| *IL-10* | Mouse | NM_010548 | F | TGC TAT GCT GCC TGC TCT TA | 243 |
|  |  |  | R | TCA TTT CCG ATA AGG CTT GG |  |
| *IL-1α* | Mouse | NM_010554 | F | GAA GCT CGT CAG GCA GAA GT | 253 |
|  |  |  | R | TGT TTC TGG CAA CTC CTT CA |  |
| *NLRP3* | Mouse | NM_145827 | F | CAG ACT GGC AAA AGG CTG TG | 333 |
|  |  |  | R | TCT TCC CGG TCT CCA TCT GT |  |
| *IL-12b* | Mouse | NM_001303244 | F | AGC AGT AGC AGT TCC CCT GA | 237 |
|  |  |  | R | TGG TTT GAT GAT GTC CCT GA |  |
| *Asc* | Mouse | NM_023258 | F | CAG AAG TGG ACG GAG TGC TG | 207 |
|  |  |  | R | AGC TCT GCT CCA GGT CCA TC |  |
| *Actin* | Mouse | NM_007393 | F | AGC CAT GTA CGT AGC CAT CC | 228 |
|  |  |  | R | CTC TCA GCT GTG GTGGTG AA |  |
| *IL-1β* | Human | NM_000576 | F | CTG TCC TGC GTG TTG AAA GA | 180 |
|  |  |  | R | TTC TGC TTG AGA GGT GCT GA |  |
| *IL-1α* | Human | NM_000575 | F | ATC AGT ACC TCA CGG CTG CT | 189 |
|  |  |  | R | TGG GTA TCT CAG GCA TCT CC |  |
| *IL-6* | Human | NM_000600 | F | TAC CCC CAG GAG AAG ATT CC | 175 |
|  |  |  | R | TTT TCT GCC AGT GCC TCT TT |  |
| *TNFα* | Human | NM_000594 | F | AAC CTC CTC TCT GCC ATC AA | 185 |
|  |  |  | R | CCA AAG TAG ACC TGC CCA GA |  |
| *NLRP3* | Human | NM_001079821 | F | GAG ACC TGT GGC CCT GGA GA | 127 |
|  |  |  | R | GGC TTG GGC CTC ATC AGA GA |  |
| *Actin* | Human | NM_001101 | F | GGA CTT CGA GCA AGA GAT GG | 234 |
|  |  |  | R | AGC ACT GTG TTG GCG TAC AG |  |
| Mouse NLRP3 promoter regions | -1,327nt | NC_000077 | F | CCA TTG TTT CTA AAG GCT TC |  |
|  | -1,216nt |  | F | CTG CCA ATC CGT CTT TGA CA |  |
|  | +166nt |  | R | CTT GAT CCA GAC GTA TGT CC |  |
